# Hydrotherapy and acupressure in restless legs syndrome: results of a randomized, controlled, three-armed, pilot study (HYDRAC-study)

**DOI:** 10.3389/fmed.2025.1571045

**Published:** 2025-04-16

**Authors:** Julia Kubasch, Miriam Ortiz, Sylvia Binting, Stephanie Roll, Katja Icke, Joanna Dietzel, Rainer Nögel, Josef Hummelsberger, Stefan N. Willich, Benno Brinkhaus, Michael Teut, Julia Siewert

**Affiliations:** ^1^Charité – Universitätsmedizin Berlin, Freie Universität Berlin and Humboldt-Universität zu Berlin, Institute of Social Medicine, Epidemiology and Health Economics, Berlin, Germany; ^2^International Society for Chinese Medicine (SMS), Munich, Germany

**Keywords:** restless legs syndrome, complementary and integrative medicine, hydrotherapy, Kneipp therapy, acupressure, randomized controlled trial

## Abstract

**Study objectives:**

Non-pharmacological interventions for restless legs syndrome (RLS) are frequently used, although scientific evidence remains limited. The study aimed to investigate the feasibility and effects of self-applied hydrotherapy and self-applied acupressure in patients with RLS.

**Methods:**

In a three-armed randomized single-center open exploratory pilot study, adults with moderate to severe RLS were randomly allocated to 6 weeks of daily hydrotherapy plus routine care (HT group), acupressure plus routine care (AP group), or routine care alone (RC group). Outcome measures included RLS symptom severity (IRLS), disease-specific quality of life (RLSQoL), the impression of change (PGI-C), health-related quality of life (SF-12), psychological outcomes (SGW-B, HADS, and GSE), and adherence and adverse events (AEs) after 6 and 12 weeks.

**Results:**

Fifty-four adults (mean age 57.5 ± 11.4 years, 63% women) were included. The study showed good feasibility with an 83% retention rate. After 6 weeks, baseline-adjusted mean IRLS scores were 19.8 (95% [16.4, 23.2]) for HT, 22.9 (19.2, 26.6) for AP, and 24.0 (20.8, 27.2) for RC. RLSQoL adjusted means were 65.3 (59.7, 70.9) for HT, 68.3 (62.3, 74.3) for AP, and 56.2 (50.9, 61.5) for RC, after 6 weeks. Both interventions were safe, with high adherence rates.

**Conclusion:**

Self-applied hydrotherapy and acupressure appear to be feasible and safe interventions for patients with RLS. This exploratory pilot study suggests potential benefits, though larger, well-designed confirmatory studies are needed to validate these findings.

**Clinical trial registration:**

This study was registered in the German Clinical Trials Register (number DRKS00029960) on August 09, 2022. https://drks.de/search/de/trial/DRKS00029960.

## 1 Introduction

Restless legs syndrome (RLS) is a common, circadian, sensorimotor disorder characterized by unpleasant sensations in the legs at rest and an urge to move them (1). Symptoms typically appear in the evening and at night, causing severe sleep disturbances and poor quality of life (2, 3). The prevalence of RLS in adults is estimated at 5.9% in Europe (4), leading to absence from work, loss of social networks, and even early retirement (5, 6), thus causing considerable costs (7). The prevalence of RLS is consistently higher in patients with a high burden of comorbidity (8).

The clinical history as the primary diagnostic method follows the five diagnostic criteria for RLS developed by the International Restless Legs Syndrome Study Group (IRLSSG) and can be obtained by any physician (8–10). The quality of life (QoL) of RLS patients is generally worse than that of individuals with type 2 diabetes, depression, and osteoarthritis (11). In comparison with control subjects without sleep disorders, RLS patients often exhibit anxiety or symptoms of depression, other psychopathological symptoms, and poor wellbeing (12). Psychological stress is known to exacerbate RLS symptoms (13, 14). RLS is a complex disorder in which dopamine and iron metabolism, predisposing genetic factors, environmental factors, and comorbidities could be involved (15).

The treatment of RLS initially focuses on iron metabolism and iron supplementation (16). Dopaminergic drugs are used if iron supplementation does not improve symptoms or the requirements are not met. Many dopaminergic drugs can cause augmentation, which is the amplification of RLS symptoms and occurs in 30–68% of patients (17). Several nondopaminergic drugs for RLS, including opioids, anticonvulsants, and alpha-adrenergic agonists (clonidine), also have common and well-known side effects that limit the effectiveness of therapy, including nausea, vomiting, urinary retention, and constipation (18–22).

Non-pharmacological interventions are often recommended as treatments for mild and intermittent RLS (16, 23, 24). However, the recommendations are generally unspecific, and to date, there are few conclusive research results, with insufficient and inconclusive evidence for many interventions requiring further research and innovation (25–27). Systematic reviews and a meta-analysis indicate that acupuncture (AC) significantly reduces RLS symptoms (25, 28–30). While AC uses needles to stimulate acupuncture points, acupressure (AP) involves manual stimulation of the same points, making it suitable for self-treatment. An AP pilot study reported a reduction in RLS severity for dialysis patients with RLS (31).

In pregnant women, cold water applications (20–25°C) may reduce RLS symptoms (32), and cold air applications (local cryotherapy [17°C] on the legs) can reduce sleep-related symptoms (25, 28, 29).

Hydrotherapy (HT) according to the German self-taught naturopath and priest Sebastian Kneipp (1821–1897) is characterized by serial, mostly cold water applications (e.g., affusions, compresses, washes, and baths) and has been known in German-speaking countries since the 19th century for preventive health care and the treatment of various diseases (33, 34). Self-applied AP and Kneipp HT showed little to no side effects in various trials (34–37). Sixty-five percent of RLS patients regularly use traditional complementary and integrative medicine (TCIM) to relieve their symptoms (38). To our knowledge, there are no randomized controlled trials that have investigated the effect, safety, and feasibility of self-applied AP and Kneipp-HT in patients with RLS.

We conducted this exploratory clinical study to evaluate the feasibility and effects of self-applied AP or HT in patients with RLS, providing preliminary data for future confirmatory trials.

## 2 Materials and methods

### 2.1 Design

This randomized, controlled, three-armed, explorative clinical trial was conducted at the outpatient department for Integrative Medicine at the German Charité Universitätsmedizin, Berlin between September 2022 and March 2023. The trial was approved by the Ethics Committee in Berlin (EA2/132/22, 12 July 2022) and followed Good Clinical Practice and the Helsinki Declaration. Prior to the study, all patients gave informed written and oral consent. The study is registered in the German Clinical Trials Register (DRKS00029960). The study design and methods were published earlier (39).

### 2.2 Patients and recruitment

Patients were recruited via digital newsletters of medical institutions, the homepage of the research institute, public transport advertising, and flyers at general practitioners’ clinics and neurological specialist practices.

The following study inclusion criteria applied: patients of all sexes, aged 18–75, with a confirmed RLS diagnosis meeting the diagnostic criteria defined by the International Restless Legs Syndrome Study Group (IRLSSG) (10), RLS-related complaints of at least 30 mm on a visual analog scale (VAS 0 mm = no complaints to 100 mm = the worst complaints possible), at least moderate RLS symptoms (IRLS – total score ≥ 11), and no planned change in medication during the study. Exclusion criteria included: indications for iron replacement therapy (except if already administered without symptom improvement or if refused by patient); regular intake of RLS-triggering medications (e.g., mirtazapine, mianserin, clozapine, olanzapine, risperidone, haloperidol, sulpiride, and promethazine); use of hydrotherapy, acupuncture, or acupressure within 4 weeks before or planned within 12 weeks after inclusion; acute SARS-CoV-2 infection or long-COVID syndrome; pregnancy or breastfeeding; serious acute/chronic organic or mental illness preventing study participation (e.g., advanced cardio/pulmonary disease NYHA/GOLD III + IV); Raynaud’s disease or advanced peripheral circulatory disorders; untreated dermatological conditions in treatment areas (e.g., severe atopic dermatitis, severe psoriasis, and large wounds); substance abuse; opioid therapy; concurrent study participation or participation within previous 3 months; and dependence on the study site (e.g., employment or any other professional or personal dependency relationship with the research institution).

### 2.3 Randomization and blinding

Randomization was performed centrally using a computer-generated randomization list (created with R software [version 4.1.2]) as block randomization with variable block length. The groups were allocated using a 1:1:1 ratio. Concealed allocation was carried out at the end of the inclusion examination by the study physician using an administrative database. The informed consent process and the assessment of inclusion and exclusion criteria were completed before the inclusion examination. Surname, given name, date of birth, and sex were entered into the administrative database by the study physician. All other personal data were filled in later by the study nurse. After a patient’s inclusion in the study, the system carried out automatic randomization and created the randomization confirmation which was initiated. Study physicians did not have access to the randomization list, which only showed one result at a time. After completing the baseline questionnaires, patients were informed of their randomization results and subsequently received training in the respective interventions. Patients and physicians were not blinded to treatment allocation. Statisticians were blinded to group allocation.

### 2.4 Study interventions and control

The 6-week intervention phase of the study was followed by a 6-week follow-up phase, during which the patients could optionally continue the learned interventions. Until the end of week 12, the control group did not receive any study intervention. After being randomized and allocated, patients received a 15-min instruction in self-therapy of acupressure (AP group) or hydrotherapy (HT group) by a study physician and were given an instruction booklet. During the following 6 weeks, the patients performed daily AP and HT at home. In both intervention groups, patients were contacted by telephone in the second and fourth weeks (w2 and w4) to inquire about difficulties with the application and to improve adherence. Discontinuations and withdrawals were documented with reasons, where known.

**Figure 1:**
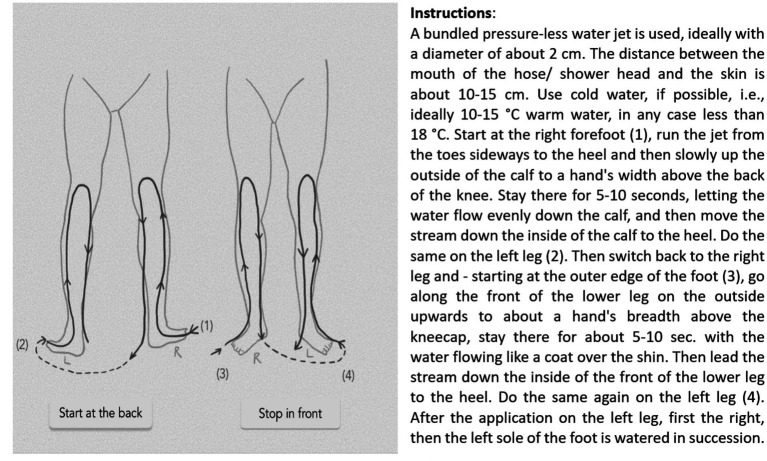
Hydrotherapy procedure for knee affusion.

Hydrotherapy was carried out in a semi-standardized manner alongside routine care with obligatory and optional affusions according to the principles of one of the pioneers of hydrotherapy, the German priest Sebastian Kneipp (1821–1897). The patients performed treatments at least twice daily for 6 weeks. Two cold affusions up to the knees daily for 30–60 s with water colder than 18°C (ideally 10–15°C) were recommended (Figure 1). The total treatment time including preparation and post-processing should take approximately 20 min daily. Optional affusions included cold or alternating warm arm or knee affusions and cold face affusions. The optional affusions could be conducted as often as desired during the day according to Kneipp’s basic rules.

**Figure 2:**
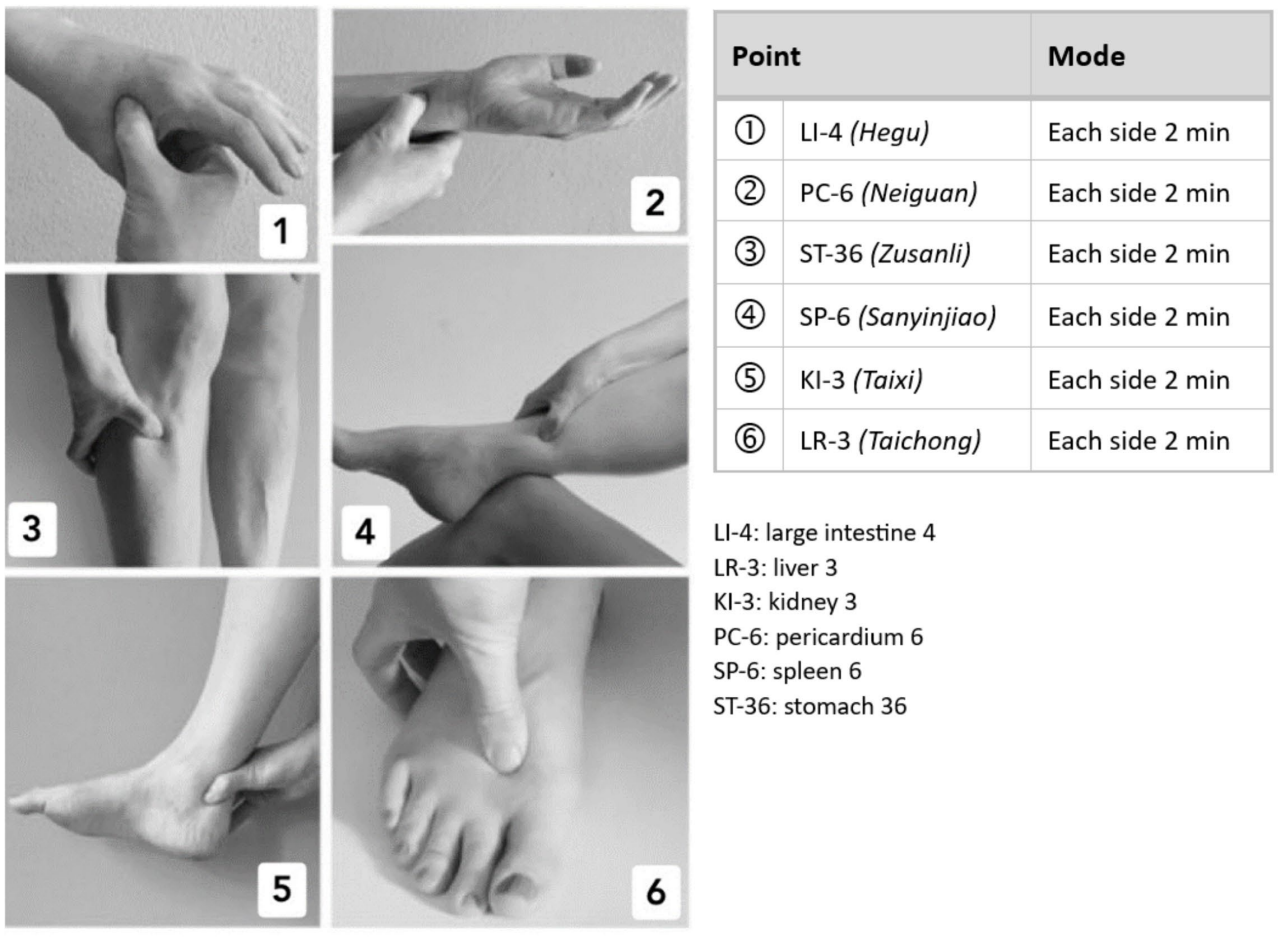
Acupressure points 1 to 6: LI 4, PC 6, ST 36, SP 6, KI 3, LR 3.

Acupressure was performed in addition to routine care by manually stimulating six acupressure points, which were determined in advance by a modified expert consensus procedure according to the rules and principles of Chinese medicine. The following points were used bilaterally: Large Intestine 4, Pericardium 6, Stomach 36, Spleen 6, Kidney 3, and Liver 3 (see Figure 2). Patients performed the treatment at least once a day for 6 weeks, or more often if desired. The total treatment time was set at approximately 20 min per day, while the pressure duration per point was approximately 2 min.

The control group was asked to continue only their routine care for 12 weeks. Details on the interventions were published before (39).

All patients were asked not to start other treatments for RLS-related symptoms during the study.

### 2.5 Outcomes

Exploratory outcome parameters were assessed after 6 and 12 weeks. Outcome parameters included RLS severity (IRLS; range 0–40, higher scores indicating more severe symptoms) (40), disease- and health-related quality of life measured with the Restless Legs Syndrome Quality of Life questionnaire (RLSQoL; range 0–100, higher scores indicating better disease-related quality of life) and the Short-Form 12 (SF-12) questionnaire yielding Physical and Mental Component Summary scores (PCS, MCS; range 0–100, higher scores indicating better health-related quality of life) (41–43), Patient Global Impressions Scale-Change (PGI-C; range 1–7, lower scores indicating greater improvement) (44), subjective global wellbeing with a 0–100 mm VAS (SGW-B; higher scores indicating better wellbeing) (45), depression and anxiety using the Hospital Anxiety and Depression Scale (HADS-D; range 0–21 for each subscale, higher scores indicating more severe symptoms) (46), and self-efficacy via the General Self-Efficacy Scale (GSE; range 10–40, higher scores indicating greater self-efficacy) (47, 48).

The minimum clinically important difference (MCID) is defined as the smallest difference between two groups on an outcome measure that is considered clinically relevant to patients.

RLS-specific MCIDs were only found for the IRLS (MCID = 3) (49). Thus, the MCID for the quality of life instrument RLSQoL was estimated based on existing evidence that MCIDs/MIDs measuring differences in quality of life are consistently close to half a standard deviation, which corresponds to 7.1% or 0.5 points on a 7-point scale (50, 51). The estimated MCID for RLSQoL is therefore 7.1 points on a respective scale of 0–100. For the SF-12, several studies were found in which the MCID was reported for patients with orthopedic conditions, obesity, and prostate cancer. The MCID for the SF-12 MCS score ranged from 1.5 to 15.9, for the PCS from 1.8 to 12 points for patients with orthopedic conditions, 4 points for MCS/PCS for patients with prostate cancer, and 5 points for MCS/PCS for patients with obesity. Based on the average of these data, we estimated the MCID of the SF-12 MCS score to be 5.9 points and that of the PCS score to be 5.3 points (52–54).

As the PGI-C is a Likert scale from 1 to 7, a difference of 0.5 points was assumed to be clinically relevant (51). The MCID for subjective global wellbeing (SGW-B), a visual analog scale (0–100 mm), was set at 14 points for our study, comparable to the MCID for the visual analog scale in the field of pain therapy. Here, differences of 14 or 30 mm on a 100 mm scale are regarded as a minimal clinically important difference (MCID) (55, 56). The MCID for the HADS-D was estimated to be 1.7 points in our study, analogous to the MCID for patients with cardiovascular disease, where it was triangulated from distribution-based, anchor-based, and Delphi-based results (57). As no MCID was found at all for the GSE, a threshold value was defined based on clinical experience. Usual threshold values here are 15% of the achieved value from the total value, which for the GSE scale (values from 10 to 40) would be a difference of 4.5 points (15% out of 30) (58).

Furthermore, the patients kept a diary during weeks 1–6 in which they recorded the frequency at which the study interventions were carried out, as well as medication changes and adverse events (AEs) (59). Patients received a second diary 12 weeks after enrolment in the study to retrospectively record safety, treatment adherence, and frequency of voluntary treatment in the follow-up phase (weeks 7–12).

Two telephone calls (w2 and w4) inquired about the feasibility of the application and were documented by the study physicians.

### 2.6 Safety

AEs and severe adverse events (SAEs) were recorded in addition to the diaries during phone calls in weeks 2 and 4. Study physicians classified adverse events as treatment-related or non-treatment-related.

### 2.7 Statistics

As this is an exploratory study, the sample size was determined primarily considering feasibility aspects. Assuming that approximately 10% of patients drop out of the study before week 6, 17 patients per group (51 randomized patients in total) were planned, which seemed logistically feasible at the study center, to obtain 15 patients per group at the end of week 6.

All data collected were analyzed descriptively: means, standard deviations, medians, and quartiles. Outcomes were analyzed using analysis of covariance (ANCOVA), depending on the scale, including the treatment group as a fixed-effect factor and the respective baseline value (where applicable) as a fixed covariate. For group comparisons, adjusted means with 95% confidence intervals are provided. *p*-values are considered exploratory without adjustment for multiple testing. Analysis was conducted on the full analysis set (FAS) defined according to the intention-to-treat principle without imputation of missing data. Analyses were performed using SPSS (IBM SPSS Statistics, version 25), R (version 4.1.2), and SAS (SAS for Windows, version 9.4).

*Post-hoc* analyses included the calculation of Cohen’s *d* effect sizes, which were not originally specified in the study protocol.

## 3 Results

**Figure 3:**
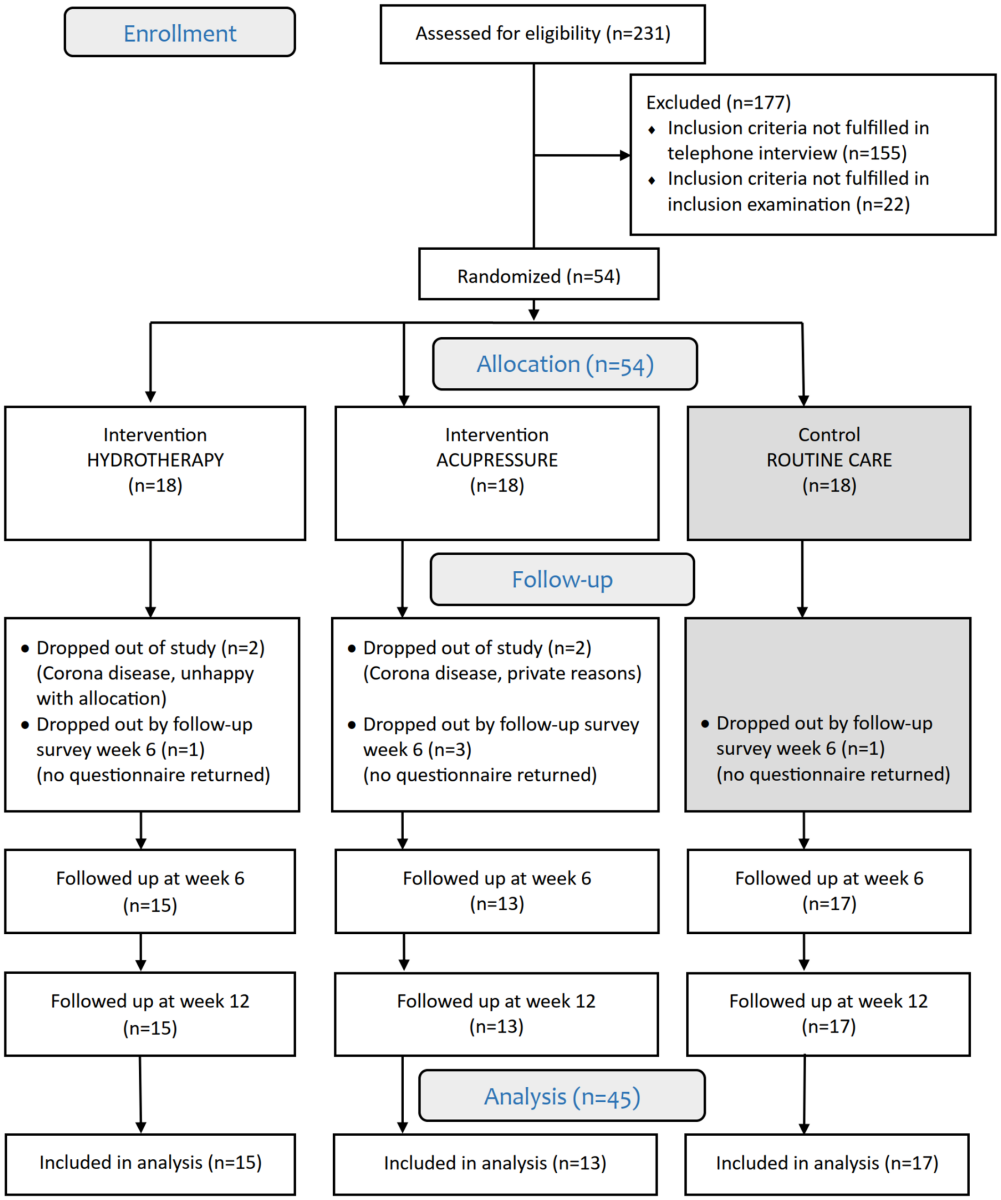
HYDRAC CONSORT study flowchart.

In total, 231 patients were screened for eligibility: 177 patients did not meet the eligibility criteria (see consort flowchart in Figure 3). The main reasons for not qualifying for participation were age over 75, the use of opioids, or only mild RLS symptoms. Fifty-four patients were included and randomly assigned to the intervention or RC group (18 patients per group). The retention rate was 83% with 45 out of 54 patients completing the 12-week study. One patient in the AP group discontinued treatment after the first week for personal reasons but continued to complete diaries and questionnaires, showing a worsening in all scores except PGI-C after 12 weeks; these data were included in the analysis.

Patient characteristics at baseline regarding age, height, weight, psychological assessment, disease-specific assessment (IRLS, RLSQoL), number of patients taking RLS medication, and average disease duration were comparable between groups (Table 1). More women were represented in the study population (63%). The sex ratio (female/male) was different between the HT group (9/9), AP group (11/7), and RC group (14/4). Compared to the intervention groups, the RC group had a higher proportion of smokers (RC: *n* = 7, AP: *n* = 4, HT: *n* = 3) and a higher level of education (RC: *n* = 13, AP: *n* = 10, HT: *n* = 9). The number of patients with concomitant diseases was high in both intervention groups. Expectations regarding the improvement potential of HT and AP did not differ between the groups and were high for both forms of therapy, although experience with both interventions was similarly low. However, the overall expectation of an improvement for AP was slightly higher than for HT, as four patients assumed that HT would not bring any improvement at all.

**Table 1:** Baseline characteristics of trial participants.

Characteristics	HT group (*n* = 18)	AP group (*n* = 18)	RC group (*n* = 18)
Age (mean [SD]), year	59.6 (7.6)	56.6 (13.3)	56.3 (12.7)
Female, *n* (%)	9 (50)	11 (61.1)	14 (77.8)
BMI (mean [SD]), kg/m^2^	25.4 (3.5)	25.5 (5.7)	25.9 (3.8)
Smokers, *n* (%)	3 (16.7)	4 (22.2)	7 (38.9)
Alcohol consumption, *n* (%)	13 (72.2)	13 (72.2)	11 (61.1)
Level of education			
Secondary school, *n* (%)	9 (50)	8 (44.4)	5 (27.8)
Higher education, *n* (%)	9 (50)	10 (55.6)	13 (72.2)
Medications^+^			
Patients taking RLS medication, *n*	11	13	12
1 RLS medication, *n*	8	10	7
2 RLS medications, *n*	2	2	3
3 or more RLS medications, *n*	1	1	2
Type of RLS medications, *n*^++^	15	18	19
Non-ergoline DA, *n*^++^	10	12	10
Levodopa, *n*^++^	4	6	6
Anticonvulsants, *n*^++^	1	0	3
Analgesic, *n*^++^	1	2	4
Antidepressant/antianxiety, *n*^++^	2	1	1
Antihypertensives, *n*^++^	8	9	3
Hormones, *n*^++^	7	3	3
Number of patients with			
No comorbid condition, *n*	3	3	5
One comorbid condition, *n*	4	8	6
Two or more comorbid conditions, *n*	11	7	7
Disease-specific assessment			
IRLS-Global Score (mean [SD])	25.4 (5.5)	22.4 (5.6)	26.1 (7.0)
RLSQoL (mean [SD])	56.0 (19.0)	56.9 (15.6)	49.9 (21.0)
Complaints due to RLS VAS (mean [SD])	61.2 (19.7)	51.4 (14.6)	63.2 (14.4)
Psychological assessment			
HADS Depression Scale (mean [SD])	7.6 (3.8)	6.6 (3.9)	7.1 (3.4)
None [0–7], *n*	9	12	10
Mild [8–10], *n*	5	4	5
Moderate [11–14], *n*	2	1	3
Severe [15–21], *n*	2	1	0
HADS Anxiety Scale (mean [SD])	9.5 (4.5)	9.1 (3.9)	10.0 (3.6)
None [0–7], *n*	7	5	4
Mild [8–10], *n*	4	5	5
Moderate [11–14], *n*	5	7	8
Severe [15–21], *n*	2	1	1
SF-12 physical component score [0–100] (mean [SD])	45.3 (8.3)	46.9 (10.0)	44.9 (7.7)
SF-12 mental component score [0–100] (mean [SD])	42.2 (11.6)	40.0 (9.9)	42.4 (12.4)
General Self-Efficacy Scale [10–40] (mean [SD])	28.6 (5.7)	28.8 (3.6)	28.3 (4.7)
Subjective Global wellbeing [VAS 0–100] (mean [SD])	52.3 (25.0)	51.9 (18.4)	51.2 (22.9)
Duration of RLS, number of patients suffering for			
1–2 years, *n*	2	0	0
2–5 years, *n*	1	3	2
More than 5 years, *n*	15	15	16
Expectations toward hydrotherapy for RLS			
No more complaints, *n*	2	0	1
Significant improvement, *n*	8	10	12
Mild improvement, *n*	6	8	3
No improvement, *n*	2	0	2
Expectations toward acupressure for RLS			
No more complaints, *n*	2	0	1
Significant improvement, *n*	9	10	10
Mild improvement, *n*	7	8	7
No improvement, *n*	0	0	0
Experiences with Hydrotherapy, *n*	1	2	3
Experiences with Acupressure [yes], *n*	1	0	1

AP, acupressure; DA, dopamine agonists; GSE [0–40], General Self-Efficacy Scale (higher values indicate a higher expectation of self-efficacy); HADS, Hospital Anxiety and Depression Scale [anxiety/depression subscales 0–21] (Higher values indicate more depressive and anxiety symptoms); HT, hydrotherapy; IRLS-Score [0–40], International RLS Severity Scale (higher scores indicate a worse outcome); RC, routine care; RLS, restless legs syndrome; RLSQoL [0–100], Restless Legs Syndrome Quality of Life questionnaire (higher values indicate a better outcome); RLS VAS [0–100], RLS Visual Analog Scale (higher values indicate more complaints); SF-12 [0–100], Short-Form-Health Survey (higher values indicate better health status); SGW-B VAS [0–100], Subjective Global Wellbeing Visual Analog Scale (higher values indicate greater wellbeing) ^+^Intake within 3 months before the start of the study; ^++^number of drugs.

**Figure 4:**
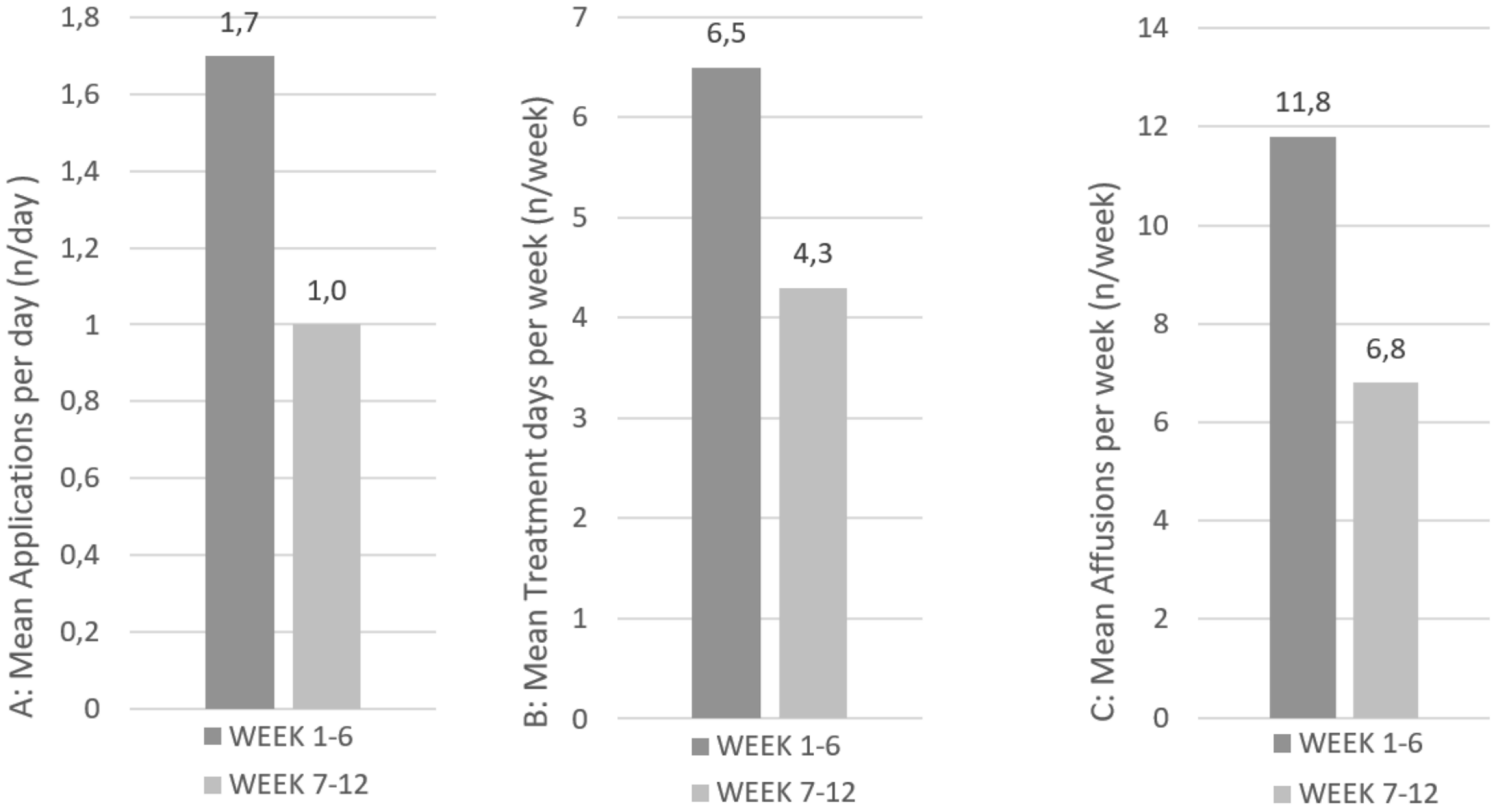
Treatment adherence of patients in the hydrotherapy group at weeks 6 and 12.

**Figure 5:**
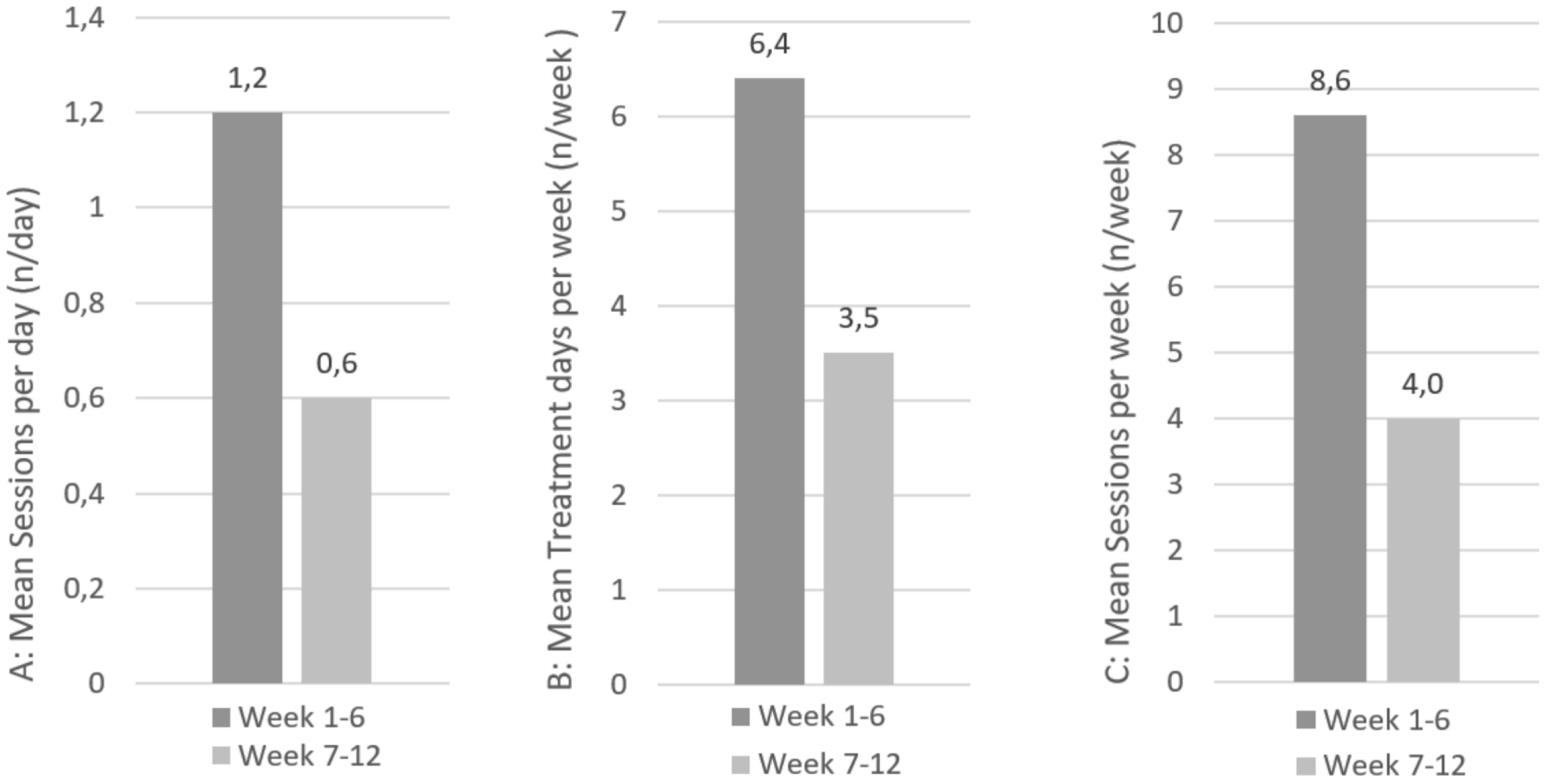
Treatment adherence of patients in the acupressure group at weeks 6 and 12.

The average intervention duration of the daily treatments differed between the intervention groups. On average, HT was performed for 5.1 min per day, while AP lasted 22.5 min per day. Adherence among the participants was similarly high in both intervention groups after 6 weeks, with self-treatment more than 6 days per week with recommended daily use (HT: 6.5, AP: 6.4 days per week, Figures 4, 5). After a 12-week follow-up, including a 6-week optional self-treatment phase, adherence remained high (HT: 4.3, AP: 3.5 days per week). During this optional self-treatment phase, a majority of patients continued their self-treatments (11/15 [73.3%] in the HT group and 10/13 [76.9%] in the AP group). After a 6-week follow-up, all participants indicated they were still motivated to continue the applications (HT: 2.2, AP: 2.5; 1 = highly motivated, 4 = no motivation). The option of additional affusions in the hydrotherapy group was used by 7 of 15 patients in the first 6 weeks. Cold arm affusions (*n* = 76, the total number of arm affusions in weeks 1–6) used by 5 of 15 patients were preferred over facial affusions (*n* = 13, the total number of face affusions in weeks 1–6) which were used by 3 of 15 patients.

**Figure 6:**
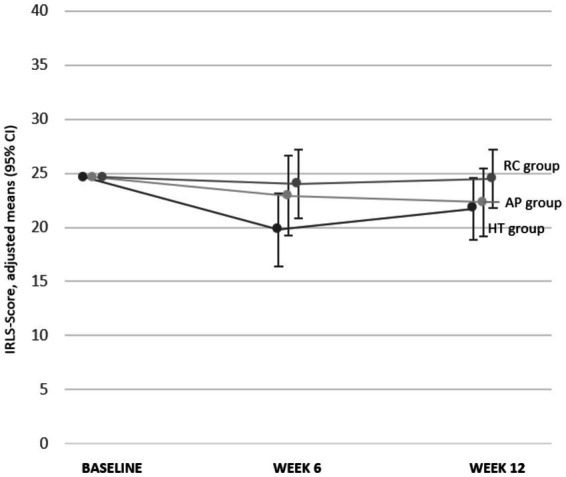
IRLS global score at baseline, at weeks 6 and 12. AP, Acupressure; CI, Confidence Interval; HT, Hydrotherapy; IRLS-Score [0–40], International RLS Severity Scale (higher scores indicate a worse outcome); RC, Routine Care.

**Figure 7:**
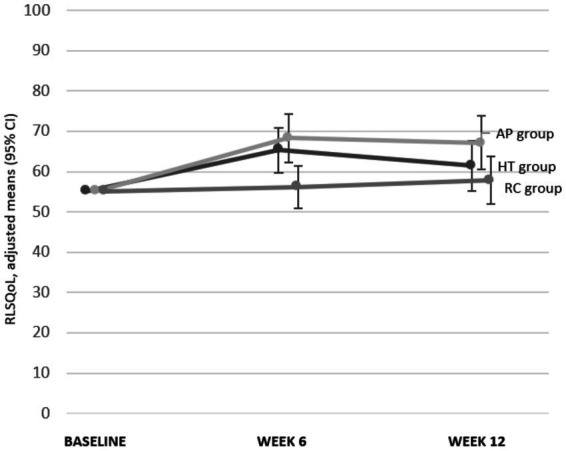
RLSQoL at baseline, at weeks 6 and 12. AP, Acupressure; CI, Confidence Interval; HT, Hydrotherapy; RLSQoL [0–100], Restless Legs Syndrome Quality of Life questionnaire (higher values indicate a better outcome).

After 6 weeks, for IRLS (MCID = 3), adjusted mean scores were 19.8 (95% CI [16.4, 23.2]; Figure 6; Table 2) for HT, 22.9 (19.2, 26.6) for AP, and 24.0 (20.8, 27.2) for RC, with mean differences compared to RC of −4.2 for HT and −1.1 for AP (negative values indicate improvement in IRLS). For RLSQoL (MCID = 7.1), adjusted means were 65.3 (59.7, 70.9) for HT, 68.3 (62.3, 74.3) for AP, and 56.2 (50.9, 61.5) for RC, with mean differences compared to RC of 9.1 for HT and 12.1 for AP (positive values indicate improvement in RLSQoL) (Figure 7; Table 2).

**Table 2:** Outcomes at week 6: group means and group differences with 95% confidence interval (CI), adjusted for respective baseline value.

	HT group (*n* = 15)	AP group (*n* = 13)	RC group (*n* = 17)	MCID	RC group vs. HT group		RC group vs. AP group		HT group vs. AP group	
	Mean (95% CI)	Mean (95% CI)	Mean (95% CI)		Mean difference (95% CI)	*p*-value	Mean difference (95% CI)	*p*-value	Mean difference (95% CI)	*p*-value
IRLS global score	19.8 (16.4; 23.2)	22.9 (19.2; 26.6)	24.0 (20.8; 27.2)	3	−4.2 (−9.8; 1.3)	0.167	−1.1 (−7.0; 4.9)	0.899	−3.1 (−9.2; 3.0)	0.432
RLSQoL	65.3 (59.7; 70.9)	68.3 (62.3; 74.3)	56.2 (50.9; 61.5)	7.1	9.1 (−0.2; 18.4)	0.056	12.1 (2.4; 21.8)	0.011	−3.0 (−12.9; 6.9)	0.740
PGI-C	3.3 (2.8; 3.8)	3.3 (2.8; 3.8)	4.1 (3.7; 4.6)	0.5	−0.8 (−1.6; 0.0)	0.062	−0.8 (−1.7; 0.0)	0.065	0.0 (−0.9; 0.9)	0.997
SF-12 PCS	47.7 (44.1; 51.3)	43.4 (39.3; 47.6)	41.0 (37.9; 44.1)	5.9	6.7 (0.9; 12.5)	0.020	2.4 (−3.9; 8.7)	0.617	4.3 (−2.5; 11.0)	0.278
SF-12 MCS	42.7 (37.3; 48.1)	41.9 (35.6; 48.1)	42.7 (38.0; 47.5)	5.3	−0.0 (−8.7; 8.7)	1.000	−0.9 (−10.3; 8.6)	0.972	0.9 (−9.2; 10.9)	0.976
SGW-B VAS	57.4 (46.5; 68.2)	54.9 (43.3; 66.6)	53.5 (43.3; 63.7)	14	3.8 (−14.1; 21.7)	0.862	1.4 (−17.3; 20.1)	0.982	2.4 (−16.8; 21.6)	0.949
HADS depression	6.5 (5.1; 8.0)	7.5 (5.9; 9.0)	7.4 (6.0; 8.7)	1.7	−0.9 (−3.2; 1.5)	0.663	0.1 (−2.4; 2.6)	0.994	−1.0 (−3.5; 1.6)	0.636
HADS anxiety	8.1 (6.5; 9.7)	8.6 (6.9; 10.3)	9.5 (8.0; 11.0)	1.7	−1.5 (−4.2; 1.2)	0.385	−0.9 (−3.7; 1.8)	0.697	−0.5 (−3.4; 2.3)	0.888
GSE	27.6 (26.0; 29.2)	26.8 (25.0; 28.6)	27.7 (26.2; 29.2)	4.5	−0.1 (−2.7; 2.5)	0.996	−0.9 (−3.6; 1.9)	0.726	0.8 (−2.0; 3.6)	0.786

AP, acupressure; CI, confidence interval; GSE, General Self-Efficacy Scale; HADS, Hospital Anxiety and Depression Scale (higher values indicate more depressive and anxiety symptoms); HT, hydrotherapy; IRLS-Score, International RLS Severity Scale (higher scores indicate a worse outcome); MCID, minimal clinically important difference; n, number; PGI-C, Patients’ Global Impression of Change (lower scores indicate a better outcome); RC, routine care; RLSQoL, Restless Legs Syndrome Quality of Life questionnaire (higher values indicate a better outcome); SF-12 PCS, Short-Form-Health Survey Physical Component Score (higher values indicate better health status), SF-12 MCS, Short-Form-Health Survey Mental Component Score (higher values indicate better health status); SGW-B VAS, Subjective Global Wellbeing Visual Analog Scale (higher values indicate greater wellbeing).

For PGI-C (MCID = 0.5), adjusted means were 3.3 (2.8, 3.8) for HT, 3.3 (2.8, 3.8) for AP, and 4.1 (3.7, 4.6) for RC, with mean differences compared to RC of −0.8 for HT and − 0.8 for AP (negative values indicate improvement in PGI-C). For SF-12 PCS (MCID = 5.9), adjusted means were 47.7 (44.1, 51.3) for HT, 43.4 (39.3, 47.6) for AP, and 41.0 (37.9, 44.1) for RC, with mean differences compared to RC of 6.7 for HT and 2.4 for AP (positive values indicate improvement in SF-12 PCS). The differences in adjusted means between groups at week 6 suggest potential clinical relevance in symptom severity for the HT group, in disease-related quality of life for both intervention groups, and in physical functioning for the HT group.

Neither of the two intervention groups showed clinically relevant differences in psychological outcomes (SGW-B VAS, HADS, GSE) or mental health-related quality of life (SF-12 MCS) compared to RC at week 6 (Table 2). The baseline values were already within the normal range or only slightly altered in all groups. However, subjective global wellbeing scores were reported as being close to the mid-level of the visual analog scale, which is consistent with a mild psychological burden in RLS patients without severe mental health impairments.

**Figure 8:**
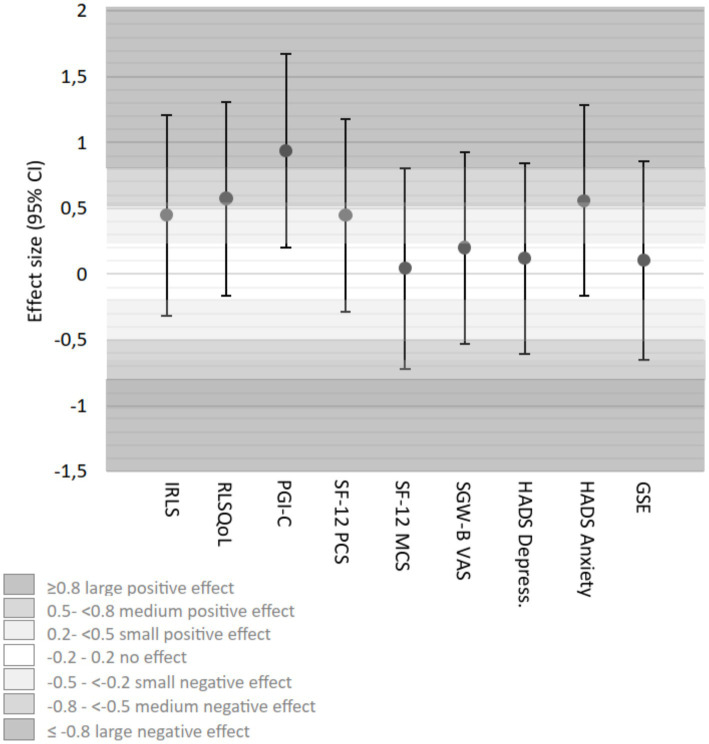
Effect sizes (Cohen’s *d*) (adjusted for baseline differences) HT group vs. RC group week 6. CI, confidence interval; GSE, General Self-Efficacy Scale; HADS, Hospital Anxiety and Depression; HT, hydrotherapy; IRLS-Score, International RLS Severity Scale; PGI-C, Patients’ Global Impression of Change; RC, routine care; RLSQoL, Restless Legs Syndrome Quality of Life questionnaire; SF-12, Short-Form-Health Survey, PCS, Physical Component Score, MCS, Mental Component Score; SGW-B VAS, Subjective Global Wellbeing Visual Analog Scale.

**Figure 9:**
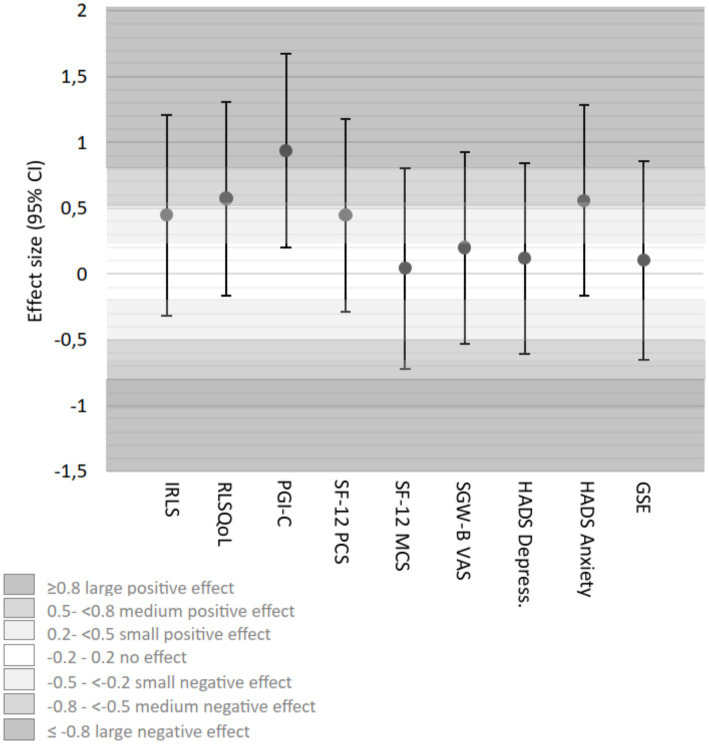
Effect sizes (Cohen’s *d*) (adjusted for baseline differences) AP group vs. RC group week 6. CI, confidence interval; GSE, General Self-Efficacy Scale; HADS, Hospital Anxiety and Depression; HT, hydrotherapy; IRLS-Score, International RLS Severity Scale; PGI-C, Patients’ Global Impression of Change; RC, routine care; RLSQoL, Restless Legs Syndrome Quality of Life questionnaire; SF-12, Short-Form-Health Survey, PCS, Physical Component Score, MCS, Mental Component Score; SGW-B VAS, Subjective Global Wellbeing Visual Analog Scale.

Effect size calculations (Cohen’s *d*) at 6 weeks showed varying magnitudes across outcomes, with positive values indicating effects in favor of the intervention (Table 3). Compared to RC, HT demonstrated small effects in IRLS (*d* = 0.44), medium effects in RLSQoL (*d* = 0.57), and large effects in PGI-C (*d* = 0.94 [Figure 8; Table 3]). AP showed medium effects in IRLS (*d* = 0.56), large effects in RLSQoL (*d* = 0.88), and medium effects in PGI-C (*d* = 0.77). Psychological outcomes showed predominantly negligible effects across all groups (Figure 9; Table 3).

**Table 3:** Effect sizes (Cohen’s *d*) group differences adjusted for baseline differences.

Outcome	RC group vs. HT group after		RC group vs. AP group after	
	6 weeks	12 weeks	6 weeks	12 weeks
IRLS	0.44	0.39	0.56	0.69
RLSQoL	0.57	0.34	0.88	0.74
PGI-C	0.94	0.73	0.77	0.26
SF-12 physical component score	0.44	0.56	0.41	0.06
SF-12 mental component score	0.04	0.55	−0.32	0.03
SGW-B VAS	0.19	0.14	0.37	−0.15
HADS depression	0.11	0.16	0.01	−0.04
HADS anxiety	0.55	0.69	0.32	0.05
GSE	0.10	−0.21	−0.21	−0.21

AP, acupressure; effect sizes (Cohen’s *d*), small (*d* ≥ 0.2), medium (*d* ≥ 0.5), and large (*d* ≥ 0.8) effect; GSE, General Self-Efficacy Scale; HADS, Hospital Anxiety and Depression Scale; HT, hydrotherapy; IRLS, International RLS Severity Scale; PGI-C, Patients’ Global Impression of Change; RC, routine care; RLSQoL, Restless Legs Syndrome Quality of Life questionnaire; SF-12, Short-Form-Health Survey; SGW-B VAS, Subjective Global Wellbeing Visual Analog Scale.

After 12 weeks, neither HT nor AP showed clinically relevant differences for IRLS compared to RC. In RLSQoL, AP continued to maintain a potential clinical benefit (MD = 9.2, 95% CI [−1.6, 20.1]) exceeding the estimated MCID of 7.1, while HT did not. PGI-C differences were no longer considered clinically relevant for either intervention group compared to RC. For SF-12 PCS and MCS, no relevant group differences were observed at 12 weeks. The psychological outcomes (SGW-B VAS, HADS, and GSE) continued to show no clinically relevant differences between the intervention groups and RC (Table 4).

**Table 4:** Outcomes at week 12: group means and group differences with 95% confidence interval (CI), adjusted for respective baseline values.

	HT group (*n* = 15)	AP group (*n* = 13)	RC group (*n* = 17)	MCID	RC group vs. HT group		RC group vs. AP group		HT group vs. AP group	
	Mean (95% CI)	Mean (95% CI)	Mean (95% CI)		Mean difference (95% CI)	*p*-value	Mean difference (95% CI)	*p*-value	Mean difference (95% CI)	*p*-value
IRLS global score	21.7 (18.9; 24.6)	22.3 (19.2; 25.5)	24.5 (21.8; 27.2)	3	−2.8 (−7.5; 1.9)	0.332	−2.2 (−7.2; 2.8)	0.543	−0.6 (−5.8; 4.6)	0.961
RLSQoL	61.4 (55.2; 67.7)	67.2 (60.5; 73.9)	58.0 (52.1; 63.8)	7.1	3.5 (−6.9; 13.9)	0.694	9.2 (−1.6; 20.1)	0.107	−5.8 (−16.8; 5.3)	0.422
PGI-C	3.5 (3.1; 4.0)	3.8 (3.3; 4.3)	4.0 (3.5; 4.4)	0.5	−0.5 (−1.2; 0.3)	0.294	−0.2 (−1.0; 0.6)	0.752	−0.2 (−1.0; 0.6)	0.749
SF-12 PCS	48.6 (45.2; 52.0)	43.1 (39.4; 46.7)	44.0 (40.9; 47.0)	5.9	4.6 (−0.8; 10.1)	0.111	−0.9 (−6.7; 4.8)	0.918	5.5 (−0.5; 11.6)	0.076
SF-12 MCS	45.7 (39.7; 51.6)	43.8 (37.4; 50.2)	41.6 (36.4; 46.9)	5.3	4.1 (−5.5; 16.6)	0.559	2.2 (−7.8; 12.2)	0.857	1.9 (−8.8; 12.6)	0.903
SGW-B VAS	59.7 (49.2; 70.2)	47.5 (36.2; 58.8)	52.5 (42.3; 62.6)	14	7.2 (−10.4; 24.8)	0.583	−5.0 (−23.3; 13.3)	0.787	12.2 (−6.4; 30.8)	0.258
HADS depression	6.2 (4.7; 7.7)	7.5 (6.0; 9.1)	7.2 (5.8; 8.6)	1.7	−1.0 (−3.4; 1.5)	0.591	0.4 (−2.2; 2.9)	0.940	−1.3 (−4.0; 1.3)	0.435
HADS anxiety	7.2 (5.5; 8.9)	9.5 (7.6; 11.3)	9.3 (7.7; 11.4)	1.7	−2.2 (−5.0; 0.7)	0.171	0.1 (−2.8; 3.1)	0.993	−2.3 (−5.3; 0.7)	0.171
GSE	26.4 (23.9; 29.0)	27.0 (24.2; 29.8)	28.4 (25.9; 30.9)	4.5	−1.9 (−6.2; 2.4)	0.522	−1.4 (−5.9; 3.1)	0.729	−0.5 (−5.1; 4.0)	0.955

AP, acupressure; CI, confidence interval; GSE, General Self-Efficacy Scale; HADS, Hospital Anxiety and Depression Scale (higher values indicate more depressive and anxiety symptoms); HT, hydrotherapy; IRLS-Score, International RLS Severity Scale (higher scores indicate a worse outcome); MCID, minimal clinically important difference; PGI-C, Patients’ Global Impression of Change (lower scores indicate a better outcome); RC, routine care; RLSQoL, Restless Legs Syndrome Quality of Life questionnaire (higher values indicate a better outcome); SF-12, Short-Form-Health Survey (higher values indicate better health status), PCS, Physical Component Score, MCS, Mental Component Score; SGW-B VAS, Subjective Global Wellbeing Visual Analog Scale (higher values indicate greater wellbeing).

Exploratory effect size analyses (Cohen’s *d*) at 12 weeks showed some changes compared to week 6 (Table 3). Compared to RC, HT maintained small effects in IRLS, showed small effects in RLSQoL, and moderate effects in PGI-C. AP maintained moderate effects for IRLS, showed moderate effects for RLSQoL, and small effects for PGI-C compared to RC. Consistent with the 6-week results, psychological outcomes showed negligible effects across all groups.

The total amount of medication taken and the percentage of patients taking medication remained quite stable over the course of the study in all groups. Thirty-six out of 54 patients (66.7%) were taking RLS medication at the start of the study (HT: *n* = 11/18, 61.1%; AP: *n* = 13/18, 72.2%; RC: *n* = 12/18, 66.7%) and 28 out of 45 patients (62.2%) at the end of weeks 6 and 12 (HT: *n* = 8/15, 53.3%; AP: *n* = 9/13, 69.2%; RC: *n* = 11/17, 64.7%).

None of the patients reported any serious adverse event (SAE) or adverse events (AEs) requiring medical treatment during the entire study period (Table 5). In the HT group, three patients reported mild treatment-related AEs including cold feet, foot and leg pain, and transient mild dizziness with pins and needles. In the AP group, six patients reported treatment-related AEs, mainly related to pressure application (pain in hands and finger joints, pain at acupressure points, cracked fingertips) and one patient reported headache while another reported short-term symptoms worsening during the first days. All reported AEs were mild and temporary.

**Table 5:** Reported adverse events and problems with intervention implementation.

Group	Treatment-related adverse events (n)	Description of treatment-related events	Non-treatment-related events (n)	Description of non-treatment-related events	Problems with intervention implementation (n)	Description of problems
HT group	3	Cold feet (1); Foot and leg pain (1); Transient mild dizziness and pins and needles in feet (1)	1	Right knee swelling after total knee arthroplasty^*^ (1)	1	Cold, unheated flat and lack of motivation to apply cold water (1)
AP group	6	Pain in hands and finger joints from pressing too hard (1); pain in thumb with restlessness in feet at kidney 3 acupoint (1); cracked fingertips (1); mild pain in tissue at acupressure points (1); short-term symptom worsening with leg pain during first days (1); headache (1)	0		3	Finding right pressure strength (1); acute cold (1); insufficient pressure duration due to pre-existing hand arthritis (1)

* Patient had undergone total knee arthroplasty 6 months earlier.

Numbers in parentheses indicate number of patients reporting each event/problem.

## 4 Discussion

Our results suggest that self-applied Kneipp hydrotherapy and acupressure are feasible. Moreover, self-applied hydrotherapy and acupressure were well-tolerated and showed high adherence overall.

With regard to the MCIDs of the outcomes both interventions suggest potentially clinically relevant differences compared to RC alone in disease-related quality of life and patients’ overall impression of change in clinical condition within the first 6 weeks. Furthermore, hydrotherapy resulted in a potentially clinically relevant difference in restless legs severity and the physical score of health-related quality of life after 6 weeks. After 12 weeks, there were still trends for clinically relevant differences in RLSQoL for the AP group. Notably, psychological outcomes remained largely unchanged across all groups, possibly due to near-normal baseline scores. As non-pharmacological treatments, hydrotherapy and acupressure could facilitate reducing or avoiding RLS medication side effects. However, given the exploratory nature and small sample size of this pilot study, these results should be interpreted as trends.

To our knowledge, this is the first RCT to assess the potential effects and feasibility of self-applied HT in the form of cold water affusions and self-applied AP in adults with RLS. Our study examined the effects on RLS symptom severity as well as on quality of life, depression/anxiety symptoms, and self-efficacy.

The strengths of the study include randomization, a high retention rate of 83%, the use of validated measurement instruments, and the development of a practical treatment manual, which is suitable for everyday clinical practice. The similarity in baseline characteristics across study groups suggests that the randomization process was effective. All participants scored in the moderate to severe range of RLS severity, with symptom scores comparable to those of participants in other RLS intervention trials (60–62). The calculation of Cohen’s *d* effect sizes, while not initially specified in our exploratory study protocol, provides additional standardized measures of the observed differences. Overall, the adverse events were mild and few in number, and patient adherence and motivation for self-application were high. The training was short and easy to conduct, and HT proved to be a time-saving option, as it only took 5 min a day to complete at home. Another positive aspect is that both interventions are low threshold, low-cost, can be used at home, and are easy to carry out.

However, due to its design, this exploratory RCT has several limitations such as the small sample size, which impairs the evaluation of effects and limits the generalizability of the study. Furthermore, generalizability is limited by the single-center design. The intervention training only took place once and implementation was subsequently only monitored by telephone twice. Therefore, the present study may have underestimated the impact of the interventions due to short training and little support for self-application. The comparability of the intervention groups is limited. Despite the prescribed 20-min daily intervention for both groups, the actual daily intervention duration differed substantially after 6 weeks (HT 5.2 min, AP 22.5 min), likely due to hydrotherapy’s integration into participants’ existing bathroom routines, eliminating anticipated preparation time. This study design cannot quantify various non-specific effects (such as placebo, nocebo, Hawthorne effect, regression to the mean, time effects, experimenter effect, response bias, and expectation effects) due to the absence of a placebo-controlled intervention. Future studies should consider a placebo-controlled arm (e.g., sham acupressure or temperature-matched water therapy) to better isolate the specific effects of hydrotherapy and acupressure. Moreover, an additional group of patients treated simultaneously with acupressure and hydrotherapy could provide valuable comparative findings. Furthermore, the lack of blinding may have introduced additional biases and influenced participants’ expectations, potentially affecting the results. Possible influences of concomitant medication and comorbidities on the course of symptom severity and patient’s conditions cannot be ruled out, although at least the amount of RLS medication was similar in all three groups and changed minimally over the course of the study.

Previous studies investigating cold water applications in pregnant women with RLS (32) and cryotherapy in patients with idiopathic RLS (63) reported statistically significant improvements in symptom severity (IRLS) within the group, which can also be considered clinically relevant. However, a group comparison was not performed, so the clinically relevant difference between the groups after the intervention cannot be assessed. Our findings with Kneipp-hydrotherapy suggested potential clinical benefits in symptom severity for the HT group and in quality of life for both intervention groups.

Additionally, our findings suggested potential clinically relevant benefits in the SF-12 Physical Component within 6 weeks of HT compared to control. Previous studies on hydrotherapy for post-polio syndrome and polyneuropathy showed either no or only minor improvements in physical functioning using the SF-36 Physical Component (34, 64, 65).

There are several possible pathways through which hydrotherapy may benefit RLS patients, although the exact mechanisms are unknown. Cold water stimuli activate the vegetative nervous system, triggering local and reflex-like effects such as local reactive hyperemia (66, 67) and a reduced sympathetic tone when repeated regularly (67). Reactive hyperemia is accompanied by an increase in oxygen concentration, muscular relaxation, and a subjective feeling of wellbeing (66–68). The suspected causes of the multifactorial development of RLS include peripheral hypoxia (69, 70), impaired microvascular blood flow (71), low oxygen partial pressure in the legs (72), increased spindle activity and muscle tone (73), and autonomic dysfunction (74–77).

Our pilot study suggested potential clinically relevant benefits in terms of disease-specific quality of life (RLSQoL) and patient’s global impression of change (PGI-C) after 6 weeks of self-applied acupressure compared to control, while effects on symptom severity (IRLS) were less pronounced. These results contrast with previous studies on acupuncture and non-self-applied acupressure, which showed greater symptom reduction: In three systematic reviews on acupuncture (25, 28, 29) and one meta-analysis of acupuncture (30), comparisons between the groups showed statistically significant differences in IRLS, which were also clinically relevant for acupuncture. Additionally, clinically relevant IRLS reductions within-group improvements were found in one RCT using a crossover design testing acupressure in hemodialysis patients with RLS (31). The differences in our findings compared to previous research regarding symptom severity may be due to the self-application method, sample size limitations, and the exploratory design of our study.

Acupressure and acupuncture activate identical acupoints, though self-applied acupressure may be limited to accessing certain potent paraspinal points, which are commonly used in both general and RLS-specific acupuncture treatments. It is assumed that the activation of the acupoint targets the autonomic nervous system to unfold its effect. This could be helpful in the treatment of RLS as we see an apparent autonomic dysfunction (74–76, 78). Acupressure stimulation regulates parasympathetic nervous system activity, which increases sleep quality by increasing autonomic responses and reducing psychological distress (79, 80). Since psychological stress can contribute to an unfavorable treatment outcome and may exacerbate symptoms in RLS patients, it must be considered in the treatment of severely affected patients. In addition, acupuncture has been shown to have anti-inflammatory effects (81) via multiple physiological pathways including the hypothalamus–pituitary–adrenal (HPA) axis (82–85), sympathetic pathways (via both sympathetic postganglionic neurons and the sympathoadrenal medullary axis) (84, 85), peripheral opioid mechanisms (86) and possibly parasympathetic cholinergic pathways (87–90). The anti-inflammatory effects of acupoint activation, which also runs through the opioid system, can be helpful in the treatment of RLS, as we observe impairment of the body’s opioid system (91, 92) and signs of increased inflammation (93–95) in RLS patients. It is also reported that acupuncture reduces oxidative stress (96, 97), which is discussed in the pathogenesis of RLS (98, 99).

The study results demonstrate the feasibility, acceptability, and potential effects of the study interventions and, in our opinion, justify larger confirmatory clinical trials. In addition to a larger sample size, future randomized clinical studies should have a multicenter design to achieve better generalizability and representativeness. Intervention groups with more detailed and longer treatment instruction, as well as personal follow-up meetings with the option of correcting the intervention’s execution could show greater intervention effects. A longer intervention and follow-up phase could provide a better assessment of long-term effects and the sustainability of the interventions. The impact of water affusions should be examined through a systematic comparison of different temperatures, application methods, and locations. In addition, objective measures such as heart rate variability and periodic leg movements can be included as study outcomes. Future studies should consider the use of acupressure devices with pressure sensors to achieve better standardization. Additionally, exploring alternative acupressure schemes, including paraspinal points, could provide further insights into RLS treatment. For future studies, we suggest considering the RLSQoL as a key outcome measure, given its clinical relevance in capturing the multifaced impact of RLS on patients’ lives.

## 5 Conclusion

Self-applied hydrotherapy and acupressure appear to be feasible and safe interventions for patients with RLS. This exploratory pilot study suggests potential benefits, though larger, well-designed confirmatory studies are needed to validate these findings.

## Data Availability

The original contributions presented in the study are included in the article/supplementary material, further inquiries can be directed to the corresponding author.

## Glossary

AC: Acupuncture
AEs: Adverse events
ANCOVA: Analysis of covariance
AP: Acupressure
CI: Confidence interval
CM: Centimeter
COVID-19: Coronavirus disease 2019
DRKS: Deutsches Register Klinischer Studien (German Clinical Trials Register)
FAS: Full analysis set
GSE: General Self-Efficacy Scale
HADS-D: Hospital Anxiety and Depression Score in German
HT: Hydrotherapy according to Kneipp
IRLSSG: International Restless Legs Syndrome Study Group
IRLS: International Restless Legs Syndrome Study Group Rating Scale
ITT: Intention-to-treat
MCID: Minimal clinically important difference
MD: Mean difference
MID: Minimal important difference
MS: Adjusted means
NYHA: New York Heart Association classification
PGI-C: Patient Global Impressions Scale – Change
QoL: Quality of life
RC: Routine care alone (control group)
RCT: Randomized controlled trial
RLS: Restless legs syndrome
RLSQoL: Restless-Legs-Syndrome Quality of Life questionnaire
SARS-COV-2: Severe acute respiratory syndrome coronavirus type 2
SGW-B: Subjective Global Wellbeing
SF-12: Short-Form (12) Health Survey
SPSS: Statistical Package for Social Sciences
TCIM: Traditional Complementary and Integrative Medicine
VAS: Visual analog scale
W: Week
Y: Year(s)
